# A cAMP phosphodiesterase is essential for sclerotia formation and virulence in *Sclerotinia sclerotiorum*

**DOI:** 10.3389/fpls.2023.1175552

**Published:** 2023-05-31

**Authors:** Yan Xu, Yilan Qiu, Yuelin Zhang, Xin Li

**Affiliations:** ^1^ Michael Smith Laboratories, University of British Columbia, Vancouver, BC, Canada; ^2^ Department of Botany, University of British Columbia, Vancouver, BC, Canada; ^3^ Department of Life Science, Hunan Normal University, Changsha, China

**Keywords:** *Sclerotinia sclerotiorum*, cAMP, cAMP phosphodiesterase, oxalic acid, host-induced gene silencing, pathogen control, next-generation sequencing, forward genetics

## Abstract

*Sclerotinia sclerotiorum* is a plant pathogenic fungus that causes white mold or stem rot diseases. It affects mostly dicotyledonous crops, resulting in significant economic losses worldwide. Sclerotia formation is a special feature of *S. sclerotiorum*, allowing its survival in soil for extended periods and facilitates the spread of the pathogen. However, the detailed molecular mechanisms of how sclerotia are formed and how virulence is achieved in *S. sclerotiorum* are not fully understood. Here, we report the identification of a mutant that cannot form sclerotia using a forward genetics approach. Next-generation sequencing of the mutant’s whole genome revealed candidate genes. Through knockout experiments, the causal gene was found to encode a cAMP phosphodiesterase (SsPDE2). From mutant phenotypic examinations, we found that SsPDE2 plays essential roles not only in sclerotia formation, but also in the regulation of oxalic acid accumulation, infection cushion functionality and virulence. Downregulation of *SsSMK1* transcripts in *Sspde2* mutants revealed that these morphological defects are likely caused by cAMP-dependent inhibition of MAPK signaling. Moreover, when we introduced HIGS construct targeting *SsPDE2* in *Nicotiana benthamiana*, largely compromised virulence was observed against *S. sclerotiorum*. Taken together, SsPDE2 is indispensable for key biological processes of *S. sclerotiorum* and can potentially serve as a HIGS target to control stem rot in the field.

## Introduction

3’,5’- cyclic adenosine monophosphate (cAMP) was first identified by Sutherland and colleagues in 1957 (Robison et al., 1971). It is a highly versatile secondary messenger playing essential roles in the regulation of many critical cellular processes across a wide range of organisms. For example, in bakers’ yeast *Saccharomyces cerevisiae*, cAMP regulates pseudohyphal morphogenesis (Pan and Heitman, 1999), mating (Arkinstall et al., 1991), glycogen utilization (Lemaire et al., 2004) and cell division cycle (Baroni et al., 1994). In mammalian cells, cAMP regulates processes including circadian clock (Fukuhara et al., 2004) and oxygen metabolism (Piccoli et al., 2006). In addition, cAMP-dependent signaling pathways are also essential for the survival and virulence of many pathogenic microbes. As an example, in *Candida albicans*, cAMP signaling cascade is involved in regulating key processes such as cell growth, filamentation, signal sensing, sexual mating and virulence (Huang et al., 2019).

Due to the important roles of cAMP, its biosynthesis and degradation must be strictly regulated to ensure a balanced signaling. cAMP is synthesized by plasma membrane-localized adenylate cyclase (AC) (Schwartz, 2001), which is activated by G protein-coupled receptors (GPCRs) in response to external signals, such as humidity, pH, etc. (Calebiro et al., 2009). Conversely, cAMP is degraded by phosphodiesterases (PDEs), cleaving the phosphodiester bond in cAMP to convert cAMP to AMP (Beavo et al., 2006). In mammals, there are over 10 different PDEs (Lugnier, 2006). Each PDE has a unique expression pattern and subcellular localization, which allows the regulation of specific cAMP and/or cGMP signaling pathways in different tissues and cell types (Degerman et al., 1997; Ekholm et al., 1997; Dousa, 1999).

In *S. cerevisiae*, PDE1 and PDE2 are the only two known PDEs with unrelated primary sequences, with low- and high-affinity respectively to their substrate. (Fujimoto et al., 1974; Sass et al., 1986; Nikawa et al., 1987). PDE1/CGS2 is the sole PDE in fission yeast *Schizosaccharomyces pombe*. Similar to *S. cerevisiae*, many phytopathogenic fungi also contain two PDEs, and PDE2 orthologs often play more crucial roles than PDE1 orthologs. For example, in grey mold pathogen *Botrytis cinerea*, deletion of *Bcpde2* results in severely compromised vegetative growth, conidiation, germination and virulence, while *Bcpde1* deletion mutant behaves like wildtype (Harren et al., 2013). Meanwhile, in rice blast fungus *Magnaporthe oryzae*, PDEH (high-affinity PDE2 ortholog) is the key regulator of cAMP and the loss of *PDEH* leads to dramatic defects in aerial hyphal growth and pathogenicity, while *pdel* (low-affinity PDE1 ortholog) mutant only shows mild defects (Ramanujam and Naqvi, 2010).

*Sclerotinia sclerotiorum* (Lib.) de Bary is a notorious soilborne plant fungal pathogen (Amselem et al., 2011; Xu et al., 2018). It has an extremely wide host range, capable of infecting over 600 plant species (Liang and Rollins, 2018), including many economically important crops such as canola, soybean, sunflower, and lettuce (Hegedus and Rimmer, 2005). The diseases caused by *S. sclerotiorum* are known as white mold or stem rot. It is a major threat to crop production worldwide, causing significant yield and quality losses (Bolton et al., 2006). *S. sclerotiorum* produces sclerotia, an overwintering structure that can survive in soil for many years (Adams and Ayers, 1979). Sclerotia can germinate carpogenically to release airborne ascospores as primary inoculum for new infections through reproductive apparatus apothecia (Erental et al., 2008). Sclerotia can also germinate as mycelia, infecting adjacent plants (Erental et al., 2008). Successful infection relies on the formation of infection cushions (also called compound appressoria), which enables penetration of plant tissues (Huang et al., 2008).

Various environmental factors, such as temperature, light and pH, affect sclerotia formation (Erental et al., 2008), along with genetic factors. The MAP kinase signaling cascades (MAPK cascades) are crucial in many cellular processes (Cargnello and Roux, 2011). They are conserved and involved in the development of *S. sclerotiorum*. *S. sclerotiorum* possesses three MAPKs (Amselem et al., 2011), with SsSMK1 being the most extensively studied. Our recent study has shown that the SsSMK1 cascade (SsSTE50-SsSTE11-SsSTE7-SsSMK1) is necessary for the development and virulence of *S. sclerotiorum* (Tian et al., 2023). However, the expression of *SsSMK1* is inversely associated with cAMP levels in *S. sclerotiorum*. Increased cAMP levels inhibit sclerotia development by interfering with *SsSMK1* transcription (Chen et al., 2004), demonstrating a negative impact of cAMP on Sclerotinia biology and MAPK signaling.

Interestingly, in *S. sclerotiorum*, cAMP seems to have diverse roles in its biology. Studies have shown that an adenylate cyclase loss-of-function mutant with cAMP synthesis deficiency, *sac1*, is impaired in growth, pathogenicity and sclerotia development (Jurick and Rollins, 2007). However, sclerotia development is inhibited when the endogenous and exogenous cAMP levels are elevated (Rollins and Dickman, 1998). The exact mechanism by which cAMP regulates key biological processes in *S. sclerotiorum* are still not fully understood. How cAMP homeostasis is controlled is also unclear.

In this study, we describe the identification of *Sspde2* from our forward genetics screen aiming to find mutants with sclerotia development defects (Xu et al., 2022). *Sspde2* mutants showed prolonged oxalic acid production, dysfunctional infection cushions, and compromised pathogenicity through cAMP-dependent MAPK inhibition. By performing host-induced gene silencing (HIGS) targeting *SsPDE2*, we observed largely compromised virulence of *S. sclerotiorum* on tobacco leaves. Thus, *SsPDE2* has the potential to be used as a HIGS target for the control of stem rot in plant hosts.

## Materials and methods

### Fungal strains and culture conditions

Fungal cultures were grown on potato dextrose agar (PDA, Shanghai Bio-way technology) at room temperature and stored on PDA slants at 4 °C or as sclerotia. For transformant screening, hygromycin B (Sigma) was added at a final concentration of 50 μg/ml. Bacteria used in this study were grown in Luria-Bertani (LB, Bio Basic) medium.

### Colony morphology and growth rate determination

All strains with different genotypes were grown on PDA plates for 2-3 days. Then, mycelial agar disks were taken from the colony margin with a sterilized pipet tip end (5 mm diameter), transferred to the center of fresh PDA plates (90 mm diameter) and incubated at room temperature. The colony diameter was measured every 12 hours until mycelia reached the edge of the petri plate. The images of colony morphology were taken 7- and 14-days post inoculation for sclerotia observation.

### Acidification assay

Fungal strains were inoculated on PDA medium supplemented with 50 mg/L bromophenol blue (BPB). OA production (media acidification) is indicated by a change in PDA-BPB medium from blue to yellow.

### Target gene knockout

The homologous recombination based method was used to generate *Sspde2* gene replacement cassette as previously described (Xu et al., 2022). All primers used for PCR are listed in **Supplementary Table S1**.

### Plant infection assays

Mycelial plugs (2 mm or 5 mm in diameter) of 2-day-old cultures were inoculated on unwounded or wounded Arabidopsis (*Arabidopsis thaliana*, ecotype Col-0) or tobacco (*Nicotiana benthamiana*) leaves placed on moistened paper towels in petri dishes. Inoculated leaves were incubated in a growth chamber (23°C; 16 h day/8 h night regime). The lesion sizes were quantified by ImageJ software. The virulence test was repeated at least three times with similar results.

### Infection cushions observation

Fresh mycelial plugs (5 mm in diameter) with growing hyphal tips were placed on the glass slides on moistened paper towels in petri dishes and incubated at 23°C for 36 h. The formation of infection cushions was monitored by a ZEISS light microscope.

### RNA extraction and RT-PCR analysis

To examine the transcripts of *SsSMK1* in WT and *Sspde2* mutants, actively growing mycelia of different genotypes were inoculated on individual PDA plates overlaid with the cellophane for 2-3 days before reaching the edge of the plates. About 100 mg of fungal hyphae from each genotype were collected, RNA was extracted using EZ-10 Spin Column RNA Mini-preps kit (Bio Basic). cDNA was generated by Easy Script™ reverse transcriptase (ABM). Real-time PCR (RT-PCR) was performed using SYBR premix kit (TaKaRa) to quantify the expression of *SsSMK1* in WT and *Sspde2* mutants. The *S. sclerotiorum* gene *ACTIN* (*Sscle14g099090*) was used as internal control to normalize the expression. RT-PCR assay was repeated twice, each with three biological replicates. Primers used for RT-PCR are listed in **Supplementary Table S1**.

### Transient expression of HIGS construct in *N. benthamiana* and *SsPDE2* expression examination in infected leaves

For HIGS vector construction, 958 bp sense fragment of *SsPDE2* was fused with intron 3 fragment of the malate synthase gene of *A. thaliana* (ms-i3; GenBank accession number AB005235), as described previously (Tinoco et al., 2010) through double-joint PCR. The fused fragments were cloned into plant expression vector pCambia1300 to generate the intermediate constructs, pCa-S-i. Then antisense fragment of *SsPDE2* were ligated into pCa-S-i vectors to create the final pCa-*PDE2*-RNAi construct. The primers used for making HIGS constructs were listed in **Supplementary Table S1**.

The binary pCa-*PDE2*-RNAi construct was introduced into *Agrobacterium tumefaciens* GV3101 through electroporation. The resulting agrobacteria with the optical density (OD_600_) at 0.8 were infiltrated into the right halves of four-week-old *N. benthamiana* leaves through the blunt tip of plastic syringes as described before (Wu et al., 2019). The empty vector (EV) pCambia1300 was infiltrated into the left halves as control. After infiltration, the plants were kept in dark for 3 days to induce the expression of the RNAi constructs. Then both left and right sides of infiltrated *N. benthamiana* leaves were inoculated with *S. sclerotiorum* WT strain 1980 to examine disease progression.

To assess trans-species RNAi, about 100 mg of necrotic tissues from infected tobacco leaves expressing EV or pCa-*PDE2*-RNAi construct were collected for total RNA extraction, cDNA generation and RT-PCR analysis as described above. *ACTIN* was used as internal control. RT-PCR assay was repeated twice, each with three biological replicates.

## Results

### A UV mutant of *S. sclerotiorum* exhibits no sclerotia, aberrant oxalic acid production and largely compromised virulence

**Figure 1:**
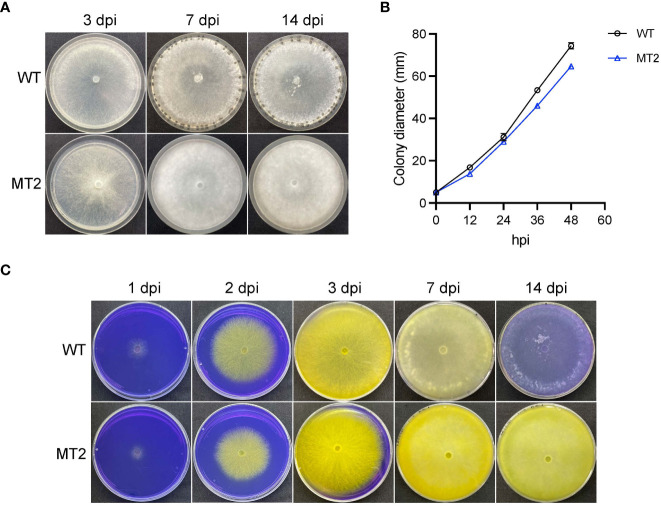
MT2 exhibits no sclerotia formation and sustained oxalic acid levels. **(A)** Colony morphology of WT and MT2. Both strains were cultured on PDA plates for 3, 7 and 14 days, respectively, before the pictures were taken. **(B)** Colony diameters of WT and MT2 on PDA plates over a period of 48 h The diameters were measured every 12 hours. **(C)** Acidification assay. Yellow color indicates acidification. Photos were taken at 1, 2, 3, 7 and 14 dpi, respectively.

We previously performed a forward genetic screen with ascospores of *S. sclerotiorum* (Xu et al., 2022). MT2 was identified as a mutant that failed to form sclerotia (**Figure 1A**). When vegetative growth was examined on petri plates, MT2 showed 13% reduction in mycelial growth as compared to wildtype (WT) strain *S. sclerotiorum* 1980 (**Figure 1B**).

To test whether MT2 is defective in pathogenicity, we first examined its oxalic acids (OA) levels, as OA secretion by Sclerotinia is a key virulence determinant of the pathogen (Cessna et al., 2000). When MT2 was grown on potato dextrose agar (PDA) supplemented with bromophenol blue (violet when pH > 4.6; yellow when pH < 3.0), both WT and MT2 plates changed color rapidly from violet to yellow within 3 dpi (days post inoculation). WT plate returned to light violet within 10 dpi, likely due to OA degradation. However, MT2 plates remained yellow even two weeks after inoculation, suggesting a sustained OA levels in MT2 (**Figure 1C**).

**Figure 2:**
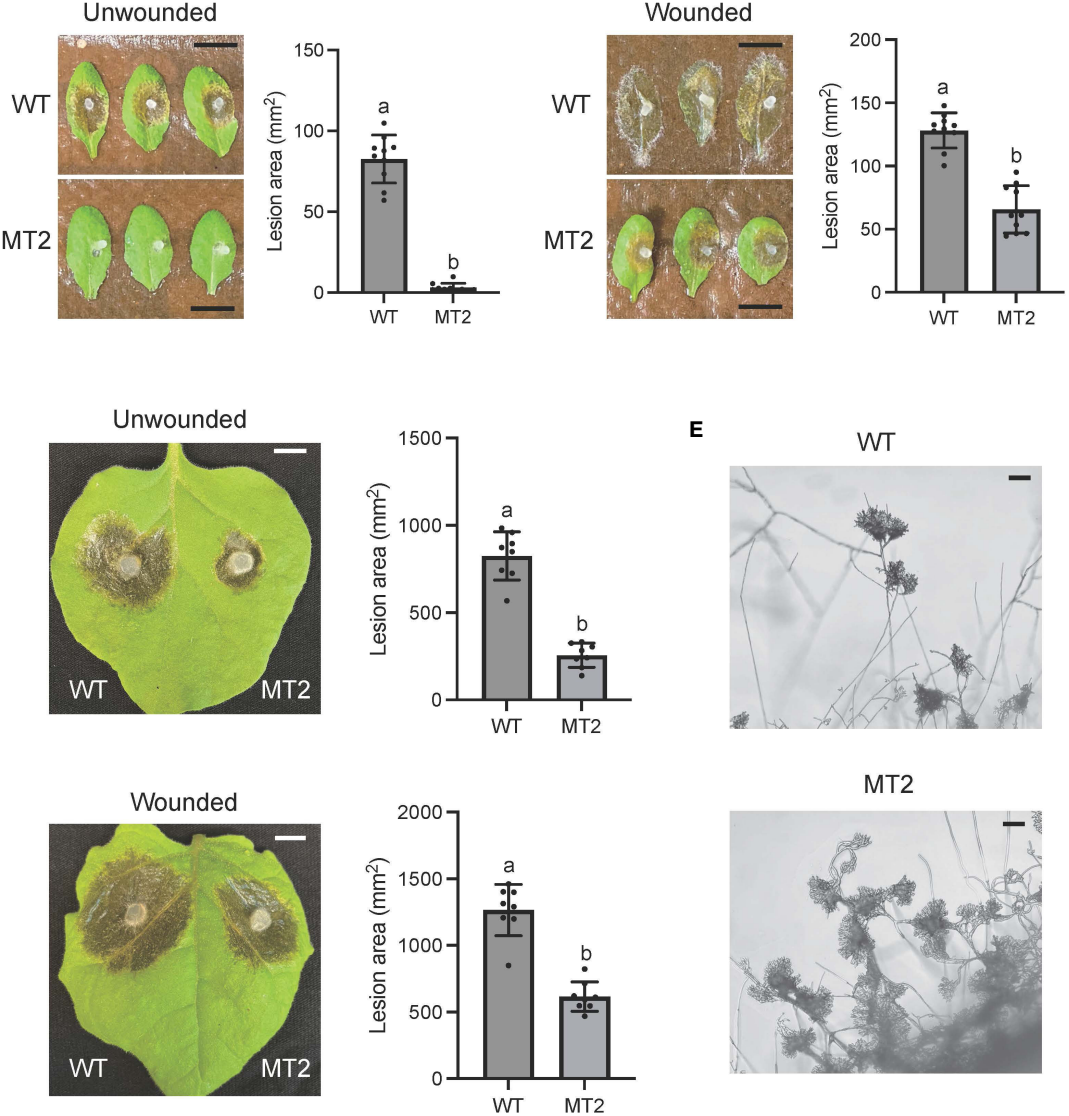
MT2 exhibits largely compromised virulence in both Arabidopsis and *N. benthamiana*. **(A, B)** Left: Pathogenicity assay for WT and MT2 on unwounded **(A)** and wounded **(B)** leaves of Arabidopsis respectively. Representative photos were taken at 36 hpi. Right: quantification of lesion areas with WT and MT2 on unwounded **(A)** and wounded **(B)** leaves of Arabidopsis respectively. The dots represent the values of lesion areas measured by ImageJ. The statistics analysis was carried out by One-way ANOVA. Letters represent statistical significance (*p* < 0.01). Error bars represent means ± Standard Deviation (SD, n= 8). The scale bar is 1 cm. (**C and D**) Left: pathogenicity assay for WT and MT2 on unwounded **(C)** and wounded **(D)** leaves of *N. benthamiana* respectively. Representative photos were taken at 48 hpi. Right: quantification of lesion areas with WT and MT2 on unwounded **(C)** and wounded **(D)** leaves of *N. benthamiana*, respectively. The dots represent the values of lesion areas measured by ImageJ. The statistics analysis was carried out by One-way ANOVA. Letters represent statistical significance (*p* < 0.01). Error bars represent means ± SD (n= 8). The scale bar is 1 cm. **(E)** Infection cushions formation on glass slides. The images were taken at 36 hpi. The scale bar is 100 μm.

To test the virulence of MT2, we used detached WT leaves of both *Nicotiana benthamiana* and Arabidopsis Col ecotype. MT2 caused no lesions when its mycelia were inoculated on Arabidopsis leaves (**Figure 2A**). It formed much smaller lesions on *N. benthimiana* (**Figure 2C**). However, with pre-wounding, the pathogenicity of MT2 on Arabidopsis and tobacco leaves was somewhat restored (**Figures 2B, D**), suggesting that the impaired pathogenicity of MT2 is due to both penetration and post-penetration defects during infection.

Infection cushion formation of *S. sclerotiorum* plays an indispensable role in its pathogenicity. WT formed mature infection cushions on glass slides within 24 hpi, shown as pigmented hyphal aggregates (**Figure 2E**). However, MT2 overproduced infection cushions. These infection structures could be observed at almost every hyphal branch tip (**Figure 2E**). Due to the penetration defects of MT2, these over-accumulated infection cushions are likely malfunctioned.

### MT2 contains a mutation in a cAMP phosphodiesterase encoding gene

**Figure 3:**
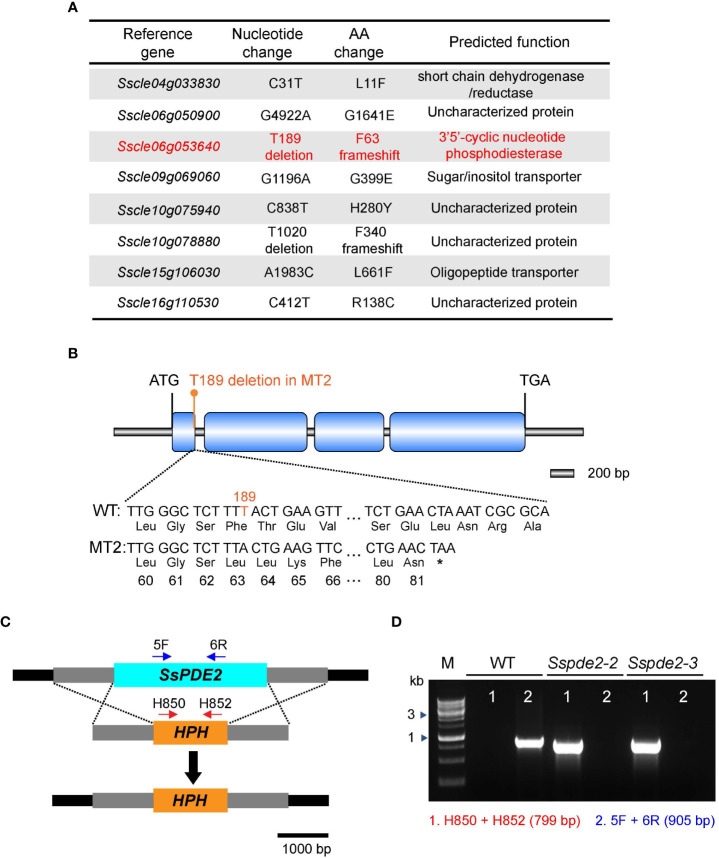
MT2 carries a mutation in *SsPDE2*. **(A)** List of candidate mutations of MT2 from NGS data analysis as compared with the reference WT *S. sclerotiorum* Strain 1980. *SsPDE2* (*Sscle06g053640*) is highlighted in red. **(B)** Diagram of the frameshift mutation in *Sscle06g053640*, including exons, introns, start codon and stop codon. The deleted nucleotide is highlighted in orange. The premature stop codon caused by the frameshift is shown as *. The diagram was drawn using Illustrator for Biological Sequencing (IBS) (Liu et al., 2015). **(C)** The *SsPDE2* locus and gene replacement design. The *SsPDE2* and *HPH* genes are presented as light blue and orange rectangles, respectively. The primers indicated by red and blue arrows in the diagram were used for knockout mutant screening. The scale is shown at the bottom. **(D)** PCR verification of *Sscle06g053640* (*SsPDE2*) gene deletion. Genomic DNA isolated from WT and mutant alleles *Sspde2-2* and *Sspde2-3* were used as PCR templates. Positions of the two pairs of primers for checking the insertion of *HPH* and *SsPDE2* deletion are indicated in **(C)** and the sizes of amplified bands are shown in brackets. M lane is DNA marker.

To identify the causal mutation responsible for the MT2 defects, the full genome of MT2 was sequenced by next -generation sequencing (NGS). Using the pipeline we established for mutation analysis (Xu et al., 2022), six significant SNPs (single nucleotide polymorphisms) leading to nonsynonymous changes were captured (**Figure 3A**). We first knocked out two candidate genes, *Sscle04g033830* and *Sscle15g106030*, and observed no phenotypic defects compared with WT (data not shown). After analyzing the NGS data for INDELs (insertion and deletion mutations), we found two of them with frameshift consequences (**Figure 3A**). One of them, *Sscle06g053640*, encodes a cAMP phosphodiesterase, SsPDE2. Previous studies in *Botrytis cinerea* showed that deletion of *Bcpde2*, the ortholog of SsPDE2, resulted in no sclerotia formation and significantly reduced virulence (Harren et al., 2013). Further analysis revealed that the frameshift mutation in *Sscle06g053640* resulted in a premature stop codon (**Figure 3B**). Considering the similar defects between MT2 and *Bcpde2*, *Sscle06g053640* became the prime candidate for MT2, and MT2 was renamed as *Sspde2-1.*

### Knocking out *SsPDE2* yielded mutants with phenotypes like MT2

A targeted gene knockout method based on homologous recombination (**Figure 3C**) and protoplast purification was used to obtain pure *Sscle06g053640* deletion mutants in the WT background. Two independent pure deletion alleles, *Sspde2-2* and *Sspde2-3*, were obtained and verified by PCR. A 905-bp fragment within the *Sscle06g053640* gene was present in WT but absent in both transformants (**Figure 3D**). The presence of selection marker gene hygromycin phosphotransferase (*HPH*) in the transformants was also confirmed by the amplification of a 799-bp product (**Figure 3D**).

**Figure 4:**
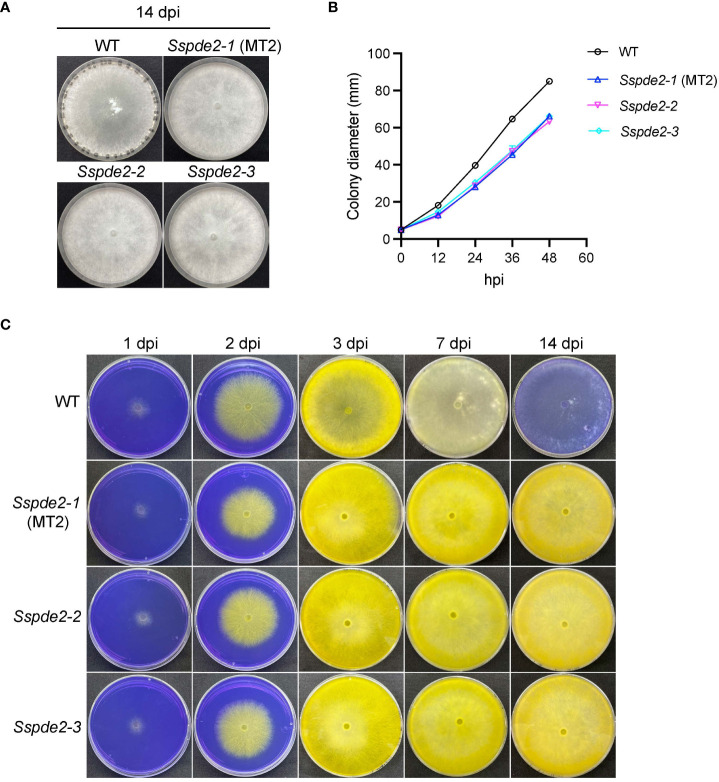
*Sspde2* deletion alleles behave similarly as MT2 in vegetative growth, sclerotia formation and OA production. **(A)** Colony morphology of WT, *Sspde2-1* (MT2), *Sspde2-2*, and *Sspde2-3*. All genotypes were cultured on PDA plates. Photos were taken at 14 dpi. **(B)** Colony diameters of the indicated genotypes on PDA plates over a period of 48 h The diameters were measured every 12 hours. **(C)** Acidification assay. Photos were taken at 1, 2, 3, 7 and 14 dpi, respectively.

**Figure 5:**
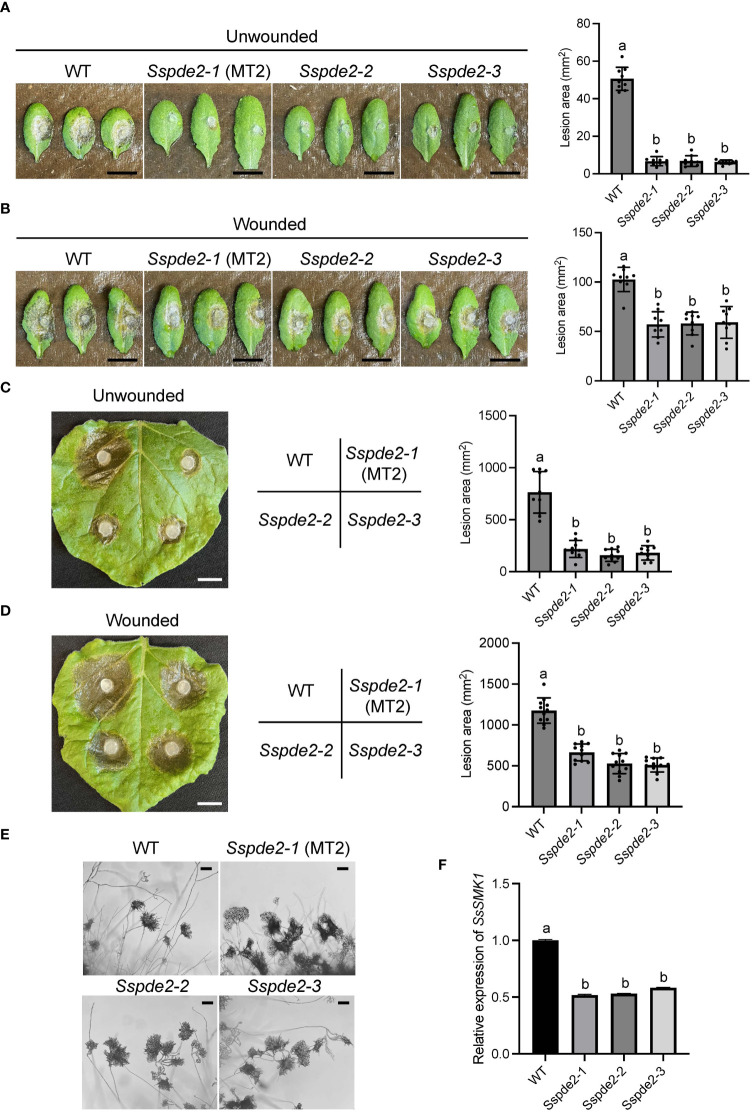
*Sspde2* deletion alleles show similar defects as MT2 in virulence, infection cushion formation and MAPK transcriptional regulation. **(A, B)** Left: Pathogenicity assay for WT, *Sspde2-1* (MT2), *Sspde2-2*, and *Sspde2-3* on unwounded **(A)** and wounded **(B)** leaves of Arabidopsis respectively. Representative photos were taken at 36 hpi. Right: quantification of lesion areas with the same sets of genotypes on unwounded **(A)** and wounded **(B)** leaves of Arabidopsis respectively. The dots represent the values of lesion areas measured by ImageJ. The statistics analysis was carried out by One-way ANOVA. Letters represent statistical significance (*p* < 0.01). Error bars represent means ± SD (n= 9). The scale bar is 1 cm. **(C, D)** Left: pathogenicity assay for WT, *Sspde2-1* (MT2), *Sspde2-2*, and *Sspde2-3* on unwounded **(C)** and wounded **(D)** leaves respectively of *N. benthamiana*. Representative photos were taken at 48 hpi. Middle: Indication of inoculated genotypes on tobacco leaf shown in the left. Right: quantification of lesion areas with the same sets of genotypes on unwounded **(C)** and wounded **(D)** leaves of *N. benthamiana*, respectively. The dots represent the values of lesion areas measured by ImageJ. The statistics analysis was carried out by One-way ANOVA. Letters represent statistical significance (*p* < 0.01). Error bars represent means ± SD (n= 9). The scale bar is 1 cm. **(E)** Infection cushions formation on glass slides. The images were taken at 36 hpi. The scale bar is 100 μm. **(F)** Relative gene expression of *SsSMK1* in WT and *Sspde2* mutants. The statistics analysis was carried out by One-way ANOVA. Letters represent statistical significance (*p* < 0.01). Error bars represent means ± SD.

When these two knockout mutants, *Sspde2-2* and *Sspde2-3*, were examined together with MT2, all displayed similar phenotypes including no sclerotia formation and slightly reduced vegetative growth (**Figures 4A, B**). Meanwhile, *Sspde2-2* and *Sspde2-3* behaved similarly as MT2 in OA accumulation (**Figure 4C**), colonization on detached leaves of Arabidopsis (**Figures 5A, B**) and *N. benthamiana* (**Figures 5C, D**) and infection cushion formation (**Figure 5E**). Therefore, we conclude that *SsPDE2* is the causal gene for the MT2 phenotypes.

### The defects of *Sspde2* are associated with cAMP-dependent MAPK inhibition

Previous study has shown that the addition of compounds which increase either endogenous or exogenous cAMP levels inhibits sclerotial development, and this cAMP-dependent inhibition is through interfering with mitogen-activated protein kinase (MAPK) signaling which regulates numerous cellular growth and developmental processes (Lewis et al., 1998). As a predicted cAMP degrading enzyme, the disruption of *SsPDE2* may lead to elevated cAMP levels, thereby blocking MAPK activation. To test this, the transcripts of MAPK signaling marker gene *SsSMK1*, an ERK-type MAPK required for sclerotial formation in *S. sclerotiorum* (Chen et al., 2004), was examined by Real time-PCR (RT-PCR). As shown in **Figure 5F**, *SsSMK1* expression was reduced by 50% in *Sspde2* mutants compared with that in WT, demonstrating that loss of *SsPDE2* is indeed associated with cAMP-mediated downregulation of MAPK activity.

### The deletion alleles of *Sspde2* failed to complement the sclerotia formation defect of MT2 by hyphal fusion

**Figure 6:**
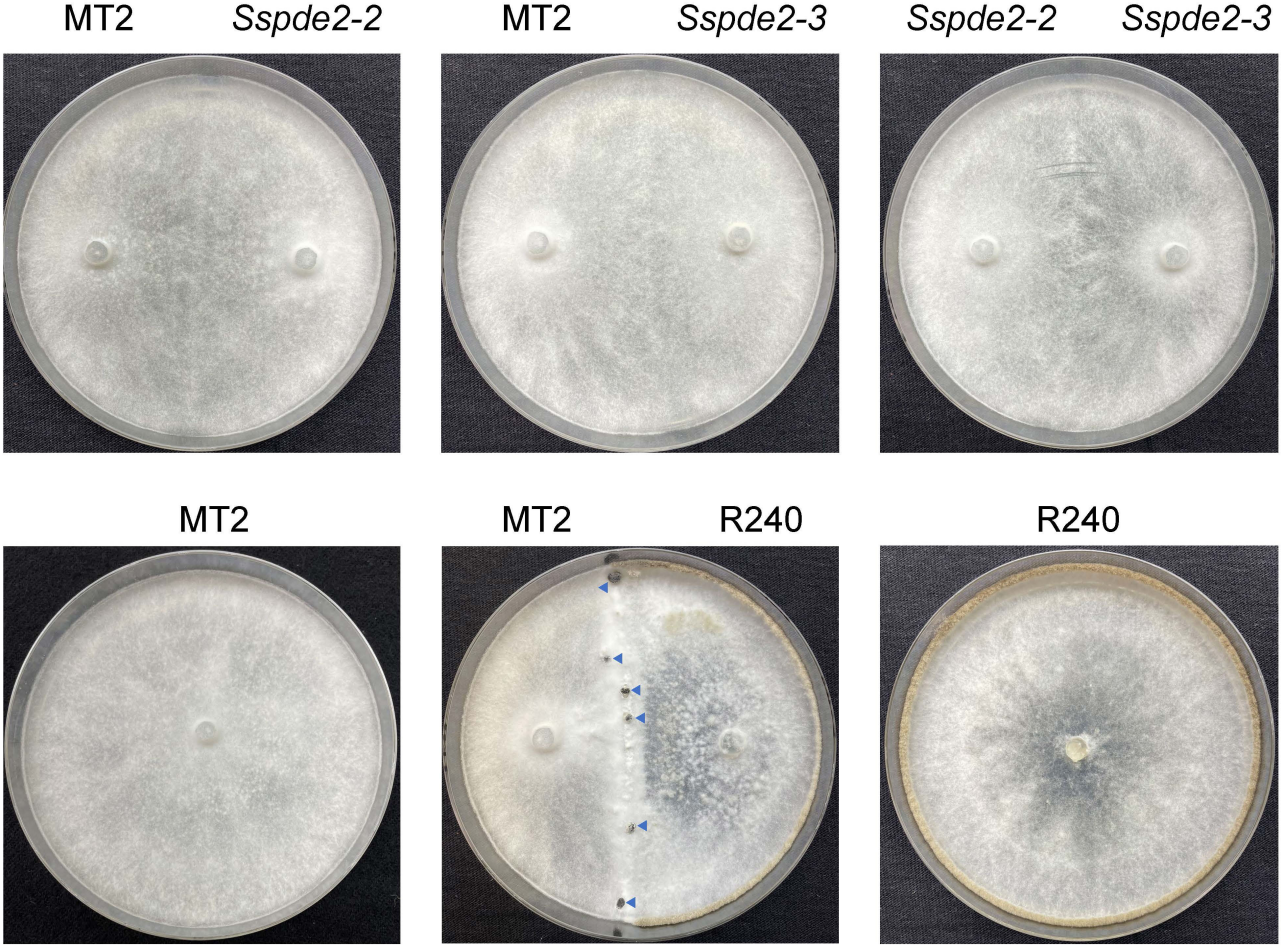
*Sspde2* deletion alleles failed to complement the sclerotia formation defect of MT2 by hyphal fusion. All genotypes with different combinations were inoculated on two sides of the plates for genetic complementation test. R240 was used as a positive control. Representative photos were taken at 7 dpi. Blue arrows indicate the sclerotia formed in the middle.

To further confirm that *SsPDE2* is responsible for the MT2 phenotypes, we performed a hyphal fusion assay. In most multinucleate fungal species, such as *Neurospora crassa* (Beadle and Coonradt, 1944) and *Aspergillus nidulans* (Timberlake and Marshall, 1988), hyphal fusion can occur between two or more genetically distinct individuals. This results in the formation of heterokaryons, which allow for genetic exchange and complementation among different nuclei within the heterokaryons. As *S. sclerotiorum* is able to form stable heterokaryons (Ford et al., 1995), mycelial fusion experiment can be performed to examine the genetic relationship between mutants. When MT2, *Sspde2-2* and *Sspde2-3* were cultured with different combinations on PDA plates for 7 days (**Figure 6**), none of these combinations gave rise to sclerotia. However, for the control plate when MT2 and R240, a non-sclerotial forming mutant found from the same screen and carries mutation in different genes (unpublished data from our lab), were fused, sclerotia formation was rescued. This suggests that *Sspde2* deletion alleles failed to complement MT2, with providing further evidence that *SsPDE2* is responsible for the MT2 phenotypes.

### SsPDE2 is predicted to be a high-affinity cAMP phosphodiesterase

**Figure 7:**
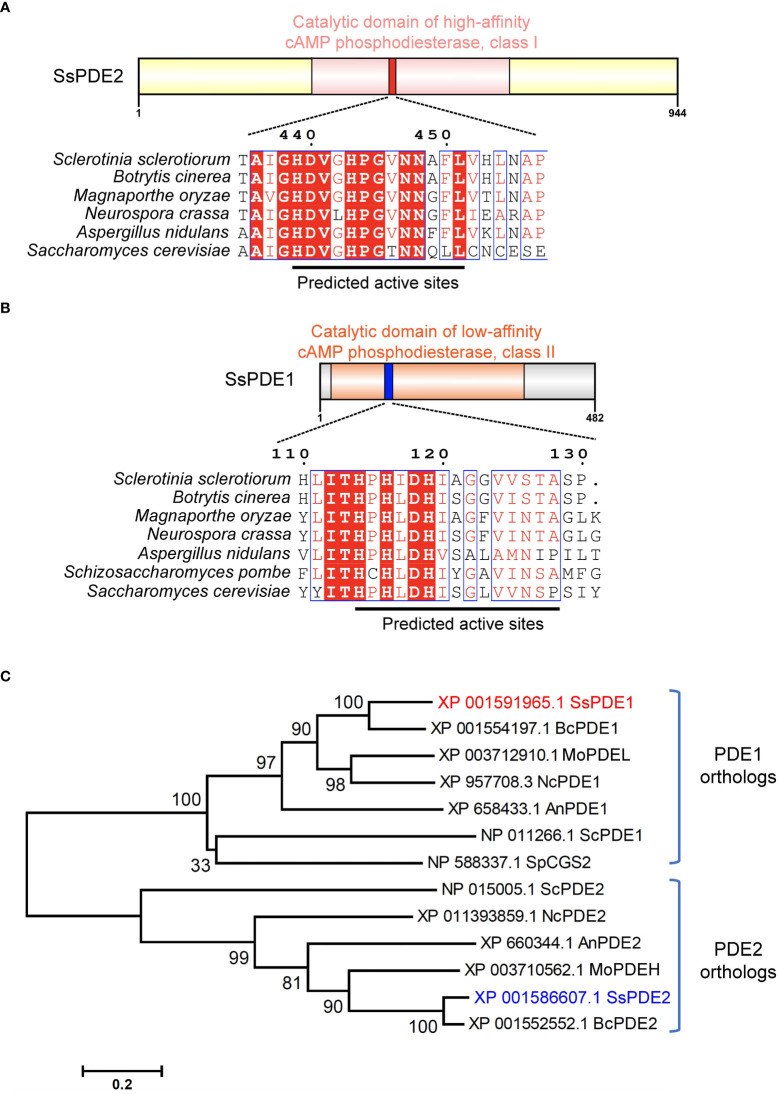
SsPDE2 is predicted to be a high-affinity cAMP phosphodiesterase. **(A, B)** Sequence analysis of SsPDE1 and SsPDE2. The catalytic domains of PDEs were analyzed by InterPro (https://www.ebi.ac.uk/interpro/). The diagrams were drawn using IBS (Liu et al., 2015). Sequence alignments of PDE1/2 orthologs in *S. sclerotiorum* and other filamentous fungi were conducted by CLUSTALW and alignment images were made using ESPript 3.0 (Robert and Gouet, 2014). Identical or conserved amino acids are either highlighted by red shadows or boxed. And the predicted PDE active sites were indicated by black lines. **(C)** Phylogenetic analysis of SsPDE1/2 proteins from *S. sclerotiorum* and other fungi. The tree was built using MEGA6, Neighbor-joining method and evaluated by bootstrap. The bootstrap values from 1,000 replicates are labelled above the branches. SsPDE1/2 are highlighted in red and blue, respectively. The accession numbers of PDEs used to build the tree are labeled on the right. The scale bar is shown at the bottom.

SsPDE2 is 944-aa in length and has a central cAMP/GMP phosphodiesterase catalytic domain (**Figure 7A**). This PDE domain shares high sequence similarity with its orthologous proteins in other filamentous fungi and all PDE2 proteins carry a conserved PDE class I motif (Ramanujam and Naqvi, 2010) at the predicted PDE active sites (**Figure 7A**). As many PDE proteins possessing class I motif have been proven to be a high-affinity cAMP PDE and control the basal levels of intracellular cAMP, such as ScPDE2 in bakers’ yeast (Sass et al., 1986) and MoPDEH in *M. oryzae* (Ramanujam and Naqvi, 2010), SsPDE2 is likely also a high-affinity PDE that can degrade cAMP and regulate cAMP levels in *S. sclerotiorum*.

Like bakers’ yeast *S. cerevisiae*, *S. sclerotiorum* also has two phosphodiesterases, PDE1 and PDE2. SsPDE1 is 482 aa in length and has a cAMP PDE catalytic domain (**Figure 7B**). Different from SsPDE2, SsPDE1 possesses a conserved class II PDE motif at the predicted PDE active sites (**Figure 7B**). Since class II PDEs have low cAMP affinity, SsPDE1 may be a low-affinity PDE in *S. sclerotiorum*.

Although both SsPDE1 and SsPDE2 are designated to be PDEs, they are dissimilar in sequences. Phylogenetic analysis of PDE1/2 orthologs in plant pathogenic fungi and yeast showed that PDE1s and PDE2s diverge early in evolution and fall into distinctive clades (**Figure 7C**). Within each clade, PDEs in pathogenic fungi are more closely related than those in yeast (**Figure 7C**), suggestive of possible conserved roles of PDEs in phytopathogenic fungi.

### HIGS of *SsPDE2* attenuates *S. sclerotiorum* virulence in tobacco

Host-induced gene silencing (HIGS) is a strategy that has been developed for plant disease control. In HIGS, plants can be engineered to express double-stranded RNAs (dsRNAs) that can target specific pathogen genes. When the pathogen is in contact with the host that can generate the dsRNAs, the dsRNAs can be up-taken by the pathogen through trans-kingdom RNAi (Wang and Dean, 2020), triggering the degradation of the corresponding target genes.

**Figure 8:**
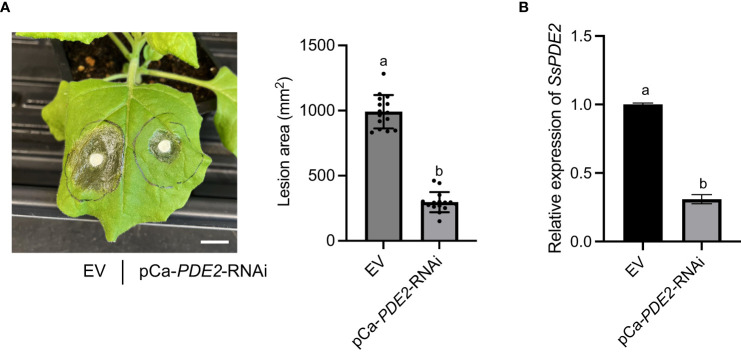
HIGS of *SsPDE2* confers resistance against *S. sclerotiorum* infections. **(A)** Left: Pathogenicity assay of *S. sclerotiorum* WT strain on leaves expressing EV (left) and pCa-*PDE2*-RNAi (right) constructs. The representative photo was taken at 48 hpi. Right: Quantification of lesion areas. The dots represent the values of lesion areas measured by ImageJ. The statistics analysis was carried out by One-way ANOVA. Letters represent statistical significance (*p* < 0.01). Error bars represent means ± SD (n= 15). The scale bar is 1 cm. **(B)** Relative gene expression of *SsPDE2* in EV and pCa-*PDE2*-RNAi expressing *N. benthamiana* leaves inoculated with *S. sclerotiorum* WT strains. The statistics analysis was carried out by One-way ANOVA. Letters represent statistical significance (*p* < 0.01). Error bars represent means ± SD.

HIGS has been shown to be effective against a wide range of plant pathogens, including fungi (Nowara et al., 2010; Song and Thomma, 2018; Spada et al., 2021) and nematodes (Iqbal et al., 2020). HIGS has been attempted in *S. sclerotiorum* previously to target well-studied pathogenicity genes, such as OA biosynthesis gene *SsOAH1* (McCaghey et al., 2021). Here, we tested whether *SsPDE2* can be used as a HIGS target. When empty vector (EV) and HIGS construct pCa-*PDE2*-RNAi were infiltrated into opposite sides of the same *N. benthamiana* leaf followed by *S. sclerotiorum* inoculation, the lesion area was largely reduced on the side expressing *SsPDE2* HIGS construct compared to the EV control (**Figure 8A**). Moreover, the gene expression level of *SsPDE2* in *S. sclerotiorum* WT strains inoculated onto *N. benthamiana* leaves expressing pCa-*PDE2*-RNAi was reduced by 70% compared with those leaves expressing EV (**Figure 8B**), demonstrating the successful trans-species RNAi of *SsPDE2*. Thus, *SsPDE2* can serve as a HIGS target for disease control against *S. sclerotiorum*.

## Discussion

In this study, we identified a putative high-affinity phosphodiesterase, SsPDE2, by a forward genetics approach. SsPDE2 is required for multiple developmental pathways and pathogenicity as mutations in *SsPDE2* result in no sclerotia formation, aberrant oxalic acid production and largely attenuated virulence in *S. sclerotiorum*.

As a predicted hydrolytic enzyme, the major biological role of PDE2 is to break down cAMP by hydrolyzing the phosphodiester bond to yield AMP (Beavo et al., 2006). Therefore, disruption of *PDE2* causes hyperaccumulation of cAMP (Park et al., 2005). Earlier studies have shown that addition of compounds which can increase cAMP levels, such as Caffeine (inhibiting phosphodiesterase activity) and NaF (activating AC), blocks scleroial initiation (Rollins and Dickman, 1998). This is consistent with our observation where *Sspde2* mutants fail to form sclerotia. Meanwhile, *Sspde2* mutants show the same colony morphology as WT *S. sclerotiorum* grown on PDA plates supplemented with 10 mM cAMP (Chen et al., 2004; Chen and Dickman, 2005). Thus, the inability in sclerotia formation in *Sspde2* is likely caused by highly elevated cAMP levels, and SsPDE2 therefore serves as a key cAMP homeostasis regulator in *S. sclerotiorum*.

In mammalian cells, cAMP binds to Rap1, a small monomeric GTPase, and inhibits the activity of MAPK cascades (Hu et al., 1997). This also occurs in *S. sclerotiorum*. It has been shown that addition of cAMP not only inhibits sclerotia formation, but also Ss*SMK1* transcription, supporting a negative role of cAMP on MAPK signaling (Chen et al., 2004; Chen and Dickman, 2005). SsSMK1 is an ERK-type MAPK that plays important role in many cellular processes in *S. sclerotiorum*. The disruption of *SsSMK1* results in reduced hyphal growth, impaired sclerotia development and attenuated pathogenicity (Chen et al., 2004; Tian et al., 2023). As *SsSMK1* downregulation was observed in *Sspde2* mutants, and considering the inhibitory effect of cAMP, it is likely that *SsPDE2* promotes *SsSMK1* transcription by reducing cellular cAMP levels.

Moreover, in this study, we found that *Sspde2* mutants exhibit prolonged acidification compared with WT. This could be ascribed to mis-regulated OA production and catabolism as it is known that increasing cellular cAMP levels enhances OA accumulation (Rollins and Dickman, 1998; Chen and Dickman, 2005). OA has been extensively studied for its role in the virulence of *S. sclerotiorum*. During the initial stages of infection, OA can act as a necrotizing virulence factor by lowering the pH of the plant tissues, assisting fungal penetration and colonization (Kim et al., 2008; Liang et al., 2015). Therefore, *S. sclerotiorum* pathogenesis requires the accumulation of high OA levels. However, in this study, sustained OA levels in *Sspde2* does not seem to aid fungal colonization. On the contrary, *Sspde2* has significantly compromised virulence. This negative effect of OA towards *S. sclerotiorum* pathogenesis can be explained by the study of an oxalate decarboxylase SsODC2, which catabolizes OA into carbon dioxide and formate. *Ssodc2* loss-of-function mutant was less efficient at compound appressorium differentiation and exhibited reduced virulence despite OA hyperaccumulation *in vitro* (Liang et al., 2015). Therefore, the role of OA in pathogenesis is complex. Proper amount of OA is required for epidermal cell disruption and penetration. When OA levels exceed certain threshold, especially with extended length of time, it may expose the fungus to toxicity, compromising the pathogen.

Like other filamentous fungi, *S. sclerotiorum* has two PDEs, SsPDE1 and SsPDE2. Their respective orthologs, BcPDE1 and BcPDE2, have been studied in another closely related sclerotia-forming pathogen *B. cinerea*. Deletion of *BcPDE2* resulted in significantly impaired hyphal growth, conidiation, spore germination, sclerotia formation and virulence. However, *bcpde1* deletion mutant is WT-like (Harren et al., 2013). Here, we found that *Sspde2* displayed similar defects as *Bcpde2* in sclerotia development and virulence, but not in vegetative growth. Only 13% growth retardation was observed in *Sspde2* compared with WT. It is possible that different from the negligible role of BcPDE1 in *B. cinerea*, SsPDE1 may contribute to the hyphal growth in *S. sclerotiorum.* This can be tested in the future by obtaining *Sspde1* mutant.

HIGS is a technique in plant disease control that involves the use of host-delivered dsRNA to silence specific genes in plant pathogens (Kong et al., 2022). HIGS can be achieved against many eukaryotic pest which has the RNAi pathway, (Shabalina and Koonin, 2008) as small interfering RNAs (siRNAs) can move from host plants to invading pathogens through cross-kingdom RNAi (Ming Wang et al., 2016). HIGS has been attempted in various plant-pathogen interactions and has been proven to be efficient in many cases (Nowara et al., 2010; Zhang et al., 2016; Spada et al., 2021). Although HIGS has the potential to be broadly applied in plant protection, one limitation is the identification of optimal targets to silence. Since our study has shown that SsPDE2 is essential for many processes in *S. sclerotiorum*, especially in virulence, silencing *SsPDE2* could potentially impair the ability of *S. sclerotiorum* to cause diseases in plants. Indeed, when we expressed cross-kingdom RNAi construct targeting *SsPDE2* in tobacco leaves, largely compromised virulence of *S. sclerotiorum* was observed. Therefore, *SsPDE2* has the potential to be used as HIGS targets for controlling stem rot in plants. Moreover, the conservation of *SsPDE2* in many necrotrophic fungal pathogens raises the possibility that *SsPDE2* may also be used to manage other diseases caused by similar fungi.

## Data availability statement

The datasets presented in this study can be found in online repositories. The names of the repository/repositories and accession number(s) can be found in the article/**Supplementary Material**.

## Author contributions

YX performed most of the experiments and wrote the manuscript draft. XL and YZ supervised the work and revised the manuscript. YQ identified the MT2 mutant in the screen. All authors contributed to the article and approved the submitted version.

## Supplementary material

Supplementary Table 1 List of primers used in this study.

## References

[B1] Adams P. B. Ayers W. A. (1979). Ecology of sclerotinia species. Phytopathology 69, 896–898. doi: 10.1094/Phyto-69-896

[B2] Amselem J. Cuomo C. A. Van Kan J. A. L. Viaud M. Benito E. P. Couloux A.. (2011). Genomic analysis of the necrotrophic fungal pathogens *Sclerotinia sclerotiorum* and *Botrytis cinerea* . PloS Genet. 7, e1002230. doi: 10.1371/journal.pgen.1002230 21876677 PMC3158057

[B3] Arkinstall S. J. Papasavvas S. G. Payton M. A. (1991). Yeast α-matching factor receptor-linked G-protein signal transduction suppresses ras-dependent activity. FEBS Lett. 284, 123–128. doi: 10.1016/0014-5793(91)80777-Z 1647971

[B4] Baroni M. D. Monti P. Lilia B. Comparata B. (1994). Repression of growth-regulated G1 cyclin expression by cyclic AMP in budding yeast. Nature 371, 339–342. doi: 10.1038/371339a0 8090203

[B5] Beadle G. W. Coonradt V. L. (1944). Heterocaryosis in *Neurospora crassa* . Genetics 29, 291–308. doi: 10.1093/genetics/29.3.291 17247122 PMC1209248

[B6] Beavo J. A. Houslay M. D. Francis S. H. (2006). Cyclic nucleotide phosphodiesterases in health and disease (CRC Press), 3–15. doi: 10.1201/9781420020847

[B7] Bolton M. D. Thomma B. P. H. J. Nelson B. D. (2006). *Sclerotinia sclerotiorum* (Lib.) de bary: biology and molecular traits of a cosmopolitan pathogen. Mol. Plant Pathol. 7, 1–16. doi: 10.1111/j.1364-3703.2005.00316.x 20507424

[B8] Calebiro D. Nikolaev V. O. Gagliani M. C. De Filippis T. Dees C. Tacchetti C.. (2009). Persistent cAMP-signals triggered by internalized G-protein-coupled receptors. PloS Biol. 7, e1000172. doi: 10.1371/journal.pbio.1000172 19688034 PMC2718703

[B9] Cargnello M. Roux P. P. (2011). Activation and function of the MAPKs and their substrates, the MAPK-activated protein kinases. Microbiol. Mol. Biol. Rev. 75, 50–83. doi: 10.1128/mmbr.00031-10 21372320 PMC3063353

[B10] Cessna S. G. Sears V. E. Dickman M. B. Low P. S. (2000). Oxalic acid, a pathogenicity factor for *Sclerotinia sclerotiorum*, suppresses the oxidative burst of the host plant. Plant Cell 12, 2191–2199. doi: 10.1105/tpc.12.11.2191 11090218 PMC150167

[B11] Chen C. Dickman M. B. (2005). cAMP blocks MAPK activation and sclerotial development via rap-1 in a PKA-independent manner in *Sclerotinia sclerotiorum* . Mol. Microbiol. 55, 299–311. doi: 10.1111/j.1365-2958.2004.04390.x 15612936

[B12] Chen C. Harel A. Gorovoits R. Yarden O. Dickman M. B. (2004). MAPK regulation of sclerotial development in *Sclerotinia sclerotiorum* is linked with pH and cAMP sensing. Mol. Plant-Microbe Interact. 17, 404–413. doi: 10.1094/MPMI.2004.17.4.404 15077673

[B13] Degerman E. Belfrage P. Manganiello V. C. (1997). Structure, localization, and regulation of cGMP-inhibited phosphodiesterase (PDE3). J. Biol. Chem. 272, 6823–6826. doi: 10.1074/jbc.272.11.6823 9102399

[B14] Dousa T. P. (1999). Cyclic-3’,5’-nucleotide phosphodiesterase isozymes in cell biology and pathophysiology of the kidney. Kidney Int. 55, 29–62. doi: 10.1046/j.1523-1755.1999.00233.x 9893113

[B15] Ekholm D. Beifrage P. Manganiello V. Degerman E. (1997). Protein kinase a-dependent activation of PDE4 (cAMP-specific cyclic nucleotide phosphodiesterase) in cultured bovine vascular smooth muscle cells. Biochim. Biophys. Acta - Mol. Cell Res. 1356, 64–70. doi: 10.1016/S0167-4889(96)00159-0 9099992

[B16] Erental A. Dickman M. B. Yarden O. (2008). Sclerotial development in *Sclerotinia sclerotiorum*: awakening molecular analysis of a ‘“ dormant “’ structure. Fungal Biol. Rev. 22, 6–16. doi: 10.1016/j.fbr.2007.10.001

[B17] Ford E. J. Miller R. V. Gray H. Sherwood J. E. (1995). Heterokaryon formation and vegetative compatibility in *Sclerotinia sclerotiorum* . Mycol. Res. 99, 241–247. doi: 10.1016/S0953-7562(09)80893-9

[B18] Fujimoto M. Ichikawa A. Tomita K. (1974). Purification and properties of adenosine 3′,5′-monophosphate phosphodiesterase from baker’s yeast. Arch. Biochem. Biophys. 161, 54–63. doi: 10.1016/0003-9861(74)90234-3

[B19] Fukuhara C. Liu C. Ivanova T. N. Chan G. C. K. Storm D. R. Iuvone P. M.. (2004). Gating of the cAMP signaling cascade and melatonin synthesis by the circadian clock in mammalian retina. J. Neurosci. 24, 1803–1811. doi: 10.1523/JNEUROSCI.4988-03.2004 14985420 PMC6730387

[B20] Harren K. Brandhoff B. Knödler M. Tudzynski B. (2013). The high-affinity phosphodiesterase BcPde2 has impact on growth, differentiation and virulence of the phytopathogenic ascomycete *Botrytis cinerea* . PloS One 8, e78525. doi: 10.1371/journal.pone.0078525 24265695 PMC3827054

[B21] Hegedus D. D. Rimmer S. R. (2005). *Sclerotinia sclerotiorum*: when “to be or not to be” a pathogen? FEMS Microbiol. Lett. 251, 177–184. doi: 10.1016/j.femsle.2005.07.040 16112822

[B22] Hu C. D. Kariya K. I. Kotani G. Shirouzu M. Yokoyama S. Kataoka T. (1997). Coassociation of Rap1A and ha-ras with raf-1 n-terminal region interferes with ras-dependent activation of raf-1. J. Biol. Chem. 272, 11702–11705. doi: 10.1074/jbc.272.18.11702 9115221

[B24] Huang L. Buchenauer H. Han Q. Zhang X. Kang Z. (2008). Ultrastructural and cytochemical studies on the infection process of *Sclerotinia sclerotiorum* in oilseed rape. J. Plant Dis. Prot. 115, 9–16. doi: 10.1007/BF03356233

[B23] Huang G. Huang Q. Wei Y. Wang Y. Du H. (2019). Multiple roles and diverse regulation of the Ras/cAMP/protein kinase a pathway in *Candida albicans* . Mol. Microbiol. 111, 6–16. doi: 10.1111/mmi.14148 30299574

[B25] Iqbal S. Fosu-Nyarko J. Jones M. G. K. (2020). Attempt to silence genes of the RNAi pathways of the root-knot nematode, meloidogyne incognita results in diverse responses including increase and no change in expression of some genes. Front. Plant Sci. 11. doi: 10.3389/fpls.2020.00328 PMC7105803 32265973

[B26] Jurick W. M. Rollins J. A. (2007). Deletion of the adenylate cyclase (sac1) gene affects multiple developmental pathways and pathogenicity in *Sclerotinia sclerotiorum* . Fungal Genet. Biol. 44, 521–530. doi: 10.1016/j.fgb.2006.11.005 17178247

[B27] Kim K. S. Min J. Y. Dickman M. B. (2008). Oxalic acid is an elicitor of plant programmed cell death during *Sclerotinia sclerotiorum* disease development. Mol. Plant-Microbe Interact. 21, 605–612. doi: 10.1094/MPMI-21-5-0605 18393620

[B28] Kong X. Yang M. Le B. H. He W. Hou Y. (2022). The master role of siRNAs in plant immunity. Mol. Plant Pathol. 23, 1565–1574. doi: 10.1111/mpp.13250 35869407 PMC9452763

[B29] Lemaire K. Van De Velde S. Van Dijck P. Thevelein J. M. (2004). Glucose and sucrose act as agonist and mannose as antagonist ligands of the G protein-coupled receptor Gpr1 in the yeast *Saccharomyces cerevisiae* . Mol. Cell 16, 293–299. doi: 10.1016/j.molcel.2004.10.004 15494315

[B30] Lewis T. S. Shapiro P. S. Ahn N. G. (1998). Signal transduction through MAP kinase cascades. Adv. Cancer Res. 74, 137–139. doi: 10.1016/s0065-230x(08)60765-4 9561267

[B32] Liang X. Moomaw E. W. Rollins J. A. (2015). Fungal oxalate decarboxylase activity contributes to *Sclerotinia sclerotiorum* early infection by affecting both compound appressoria development and function. Mol. Plant Pathol. 16, 825–836. doi: 10.1111/mpp.12239 25597873 PMC6638544

[B33] Liang X. Rollins J. A. (2018). Mechanisms of broad host range necrotrophic pathogenesis in *Sclerotinia sclerotiorum* . Phytopathology 108, 1128–1140. doi: 10.1094/PHYTO-06-18-0197-RVW 30048598

[B34] Liu W. Xie Y. Ma J. Luo X. Nie P. Zuo Z.. (2015). IBS: an illustrator for the presentation and visualization of biological sequences. Bioinformatics 31, 3359–3361. doi: 10.1093/bioinformatics/btv362 26069263 PMC4595897

[B35] Lugnier C. (2006). Cyclic nucleotide phosphodiesterase (PDE) superfamily: a new target for the development of specific therapeutic agents. Pharmacol. Ther. 109, 366–398. doi: 10.1016/j.pharmthera.2005.07.003 16102838

[B36] McCaghey M. Shao D. Kurcezewski J. Lindstrom A. Ranjan A. Whitham S. A.. (2021). Host-induced gene silencing of a *Sclerotinia sclerotiorum* oxaloacetate acetylhydrolase using bean pod mottle virus as a vehicle reduces disease on soybean. Front. Plant Sci. 12. doi: 10.3389/fpls.2021.677631 PMC8329588 34354721

[B37] Nikawa J. Sass P. Wigler M. (1987). Cloning and characterization of the low-affinity cyclic AMP phosphodiesterase gene of *Saccharomyces cerevisiae* . Mol. Cell. Biol. 7, 3629–3636. doi: 10.1128/mcb.7.10.3629-3636.1987 2824992 PMC368017

[B38] Nowara D. Schweizer P. Gay A. Lacomme C. Shaw J. Ridout C.. (2010). HIGS: host-induced gene silencing in the obligate biotrophic fungal pathogen *Blumeria graminis* . Plant Cell 22, 3130–3141. doi: 10.1105/tpc.110.077040 20884801 PMC2965548

[B39] Pan X. Heitman J. (1999). Cyclic AMP-dependent protein kinase regulates pseudohyphal differentiation in *Saccharomyces cerevisiae* . Mol. Cell. Biol. 19, 4874–4887. doi: 10.1128/mcb.19.7.4874 10373537 PMC84286

[B40] Park J. I. Grant C. M. Dawes I. W. (2005). The high-affinity cAMP phosphodiesterase of *Saccharomyces cerevisiae* is the major determinant of cAMP levels in stationary phase: involvement of different branches of the ras-cyclic AMP pathway in stress responses. Biochem. Biophys. Res. Commun. 327, 311–319. doi: 10.1016/j.bbrc.2004.12.019 15629464

[B41] Piccoli C. Scacco S. Bellomo F. Signorile A. Iuso A. Boffoli D.. (2006). cAMP controls oxygen metabolism in mammalian cells. FEBS Lett. 580, 4539–4543. doi: 10.1016/j.febslet.2006.06.085 16870178

[B42] Ramanujam R. Naqvi N. I. (2010). PdeH, a high-affinity camp phosphodiesterase, is a key regulator of asexual and pathogenic differentiation in *Magnaporthe oryzae* . PloS Pathog. 6, 1–23. doi: 10.1371/journal.ppat.1000897 PMC2865543 20463817

[B43] Robert X. Gouet P. (2014). Deciphering key features in protein structures with the new ENDscript server. Nucleic Acids Res. 42, 320–324. doi: 10.1093/nar/gku316 PMC4086106 24753421

[B44] Robison G. A. Butcher R. W. Sutherland E. W. (1971). Cyclic AMP (New York: Acad. Press), 17–46. doi: 10.1016/b978-0-12-590450-6.50006-9

[B45] Rollins J. A. Dickman M. B. (1998). Increase in endogenous and exogenous cyclic AMP levels inhibits sclerotial development in *Sclerotinia sclerotiorum* . Appl. Environ. Microbiol. 64, 2539–2544. doi: 10.1128/aem.64.7.2539-2544.1998 9647827 PMC106423

[B46] Sass P. Field J. Nikawa J. Toda T. Wigler M. (1986). Cloning and characterization of the high-affinity cAMP phosphodiesterase of *Saccharomyces cerevisiae* . Proc. Natl. Acad. Sci. U. S. A. 83, 9303–9307. doi: 10.1073/pnas.83.24.9303 3025832 PMC387126

[B47] Schwartz J. H. (2001). The many dimensions of cAMP signaling. Proc. Natl. Acad. Sci. U. S. A. 98, 13482–13484. doi: 10.1073/pnas.251533998 11717418 PMC61065

[B48] Shabalina S. A. Koonin E. V. (2008). Origins and evolution of eukaryotic RNA interference. Trends Ecol. Evol. 23, 578–587. doi: 10.1016/j.tree.2008.06.005 18715673 PMC2695246

[B49] Song Y. Thomma B. P. H. J. (2018). Host-induced gene silencing compromises verticillium wilt in tomato and arabidopsis. Mol. Plant Pathol. 19, 77–89. doi: 10.1111/mpp.12500 27749994 PMC6638114

[B50] Spada M. Pugliesi C. Fambrini M. Pecchia S. (2021). Silencing of the Slt2-type MAP kinase Bmp3 in *Botrytis cinerea* by application of exogenous dsRNA affects fungal growth and virulence on *Lactuca sativa* . Int. J. Mol. Sci. 22, 5362. doi: 10.3390/ijms22105362 34069750 PMC8161090

[B51] Tian L. Li J. Xu Y. Qiu Y. Li X. (2023). A MAP kinase cascade broadly regulates development and virulence of sclerotinia sclerotiorum and can be targeted by HIGS for disease control. doi: 10.1101/2023.03.01.530680 38149487

[B52] Timberlake W. E. Marshall M. A. (1988). Genetic regulation of development in *Aspergillus nidulans* . Trends Genet. 4, 162–169. doi: 10.1016/0168-9525(88)90022-4 3076298

[B53] Tinoco M. L. P. Dias B. B. A. Dall’Astta R. C. Pamphile J. A. Aragão F. J. L. (2010). *In vivo* trans-specific gene silencing in fungal cells by in planta expression of a double-stranded RNA. BMC Biol. 8, 27. doi: 10.1186/1741-7007-8-27 20356372 PMC2907587

[B54] Wang M. Dean R. A. (2020). Movement of small RNAs in and between plants and fungi. Mol. Plant Pathol. 21, 589–601. doi: 10.1111/mpp.12911 32027079 PMC7060135

[B55] Wang M. Weiberg A. Lin F. M. Thomma B. P. H. J. Huang H. Jin H. (2016). Bidirectional cross-kingdom RNAi and fungal uptake of external RNAs confer plant protection. Nat. Plants 2, 16151. doi: 10.1038/nplants.2016.151 27643635 PMC5040644

[B56] Wu Z. Li M. Dong O. X. Xia S. Liang W. Bao Y.. (2019). Differential regulation of TNL-mediated immune signaling by redundant helper CNLs. New Phytol. 222, 938–953. doi: 10.1111/nph.15665 30585636

[B58] Xu Y. Ao K. Tian L. Qiu Y. Huang X. Liu X.. (2022). A forward genetic screen in *Sclerotinia sclerotiorum* revealed the transcriptional regulation of its sclerotial melanization pathway. Mol. Plant-Microbe Interact. 35, 244–256. doi: 10.1094/MPMI-10-21-0254-R 34813706

[B57] Xu L. Li G. Jiang D. Chen W. (2018). *Sclerotinia sclerotiorum*: an evaluation of virulence theories. Annu. Rev. Phytopathol. 56, 311–338. doi: 10.1146/annurev-phyto-080417-050052 29958073

[B59] Zhang T. Jin Y. Zhao J. H. Gao F. Zhou B. J. Fang Y. Y.. (2016). Host-induced gene silencing of the target gene in fungal cells confers effective resistance to the cotton wilt disease pathogen *Verticillium dahliae* . Mol. Plant 9, 939–942. doi: 10.1016/j.molp.2016.02.008 26925819

