# Supplementary material for: A cAMP phosphodiesterase is essential for sclerotia formation and virulence in Sclerotinia sclerotiorum
Source: Front Plant Sci. 2023 May 31;14:1175552. doi: 10.3389/fpls.2023.1175552 (PMC10264682; doi:10.3389/fpls.2023.1175552)
Supplement: Supplementary Table 1 — List of primers used in this study. [file Table_1.pdf]

Supplementary Table 1. Summary of primers.

Primer name	DNA sequence (5' to 3')	Function
SsPDE2-1F	GACACTTATACACACACACG	*SsPDE2* knockout
SsPDE2-2R	cgggtaccgagctctttgaaggttgtgGCTGAATTCTCCAAGGTCGA	*SsPDE2* knockout
SsPDE2-3F	gccgaccgggatccacttaacgttacCCTAGTCAACTTCAACTGAG	*SsPDE2* knockout
SsPDE2-4R	TTCTTCTTTCCCTCCTCCAC	*SsPDE2* knockout
SsPDE2-5F	CCACAGATTGATGGTATCCT	*SsPDE2* knockout
SsPDE2-6R	GATTCCCCAATCGTACGAG	*SsPDE2* knockout
SsPDE2-7F	CCTTCACTTAACCCATTTCC	*SsPDE2* knockout
SsPDE2-8R	CCTCTACTCAACTGATTAAC	*SsPDE2* knockout
HYG-F	CACAACCTTCAAAGAGCTCGGTACCCG	*SsPDE2* knockout
HYG-R	GTAACGTTAAGTGGATCCCGGTCGGC	*SsPDE2* knockout
H850	GTCAAGCAAGGTAAGTGAACG	*SsPDE2* knockout
H852	ATGTTGGCGACCTCGTATTGG	*SsPDE2* knockout
H855-F	GTCGATGCGACGCAATCGT	*SsPDE2* knockout
H855-R	GAACCATCTTGTCAAACGAC	*SsPDE2* knockout
ms-i3-F	GTCCTCGAAATCAGCCTGTTCA	HIGS
ms-i3-R(SpeI)	GGACTAGT TTTATGGTCCATTTTC	HIGS
PDE2-sense-F(KpnI)	CGGGGTACCCATGCAATATTGTCTACGTGG	HIGS
PDE2-sense-R	TGAACAGGCTGATTTCGAGGACGGTAAAGAGAGTGCATGTTG	HIGS
PDE2-antisense-F(SpeI)	GGACTAGTGGTAAAGAGAGTGCATGTTG	HIGS
PDE2-antisense-R(SacI)	CGAGCTCCATGCAATATTGTCTACGTGG	HIGS
SMK1-F	GTGTTGTTTGTTCAGCTTTGCA	Gene expression

SMK1-R	TGGAGATGATGTTCTCATGATTG	Gene expression
PDE-F	GGCTCTTTTACTGAAGTTCACGTTT	Gene expression
PDE-R	AACGAGGGTAGGTATCAAATCG	Gene expression
ACTIN-F	GAGCTGTTTTCCCTTCCATTGTC	Gene expression
ACTIN-R	GACGACACCGTGCTCGATTGG	Gene expression
